# Supplementary material for: Application of a life table approach to assess duration of BNT162b2 vaccine-derived immunity by age using COVID-19 case surveillance data during the Omicron variant period
Source: PLoS One. 2023 Sep 20;18(9):e0291678. doi: 10.1371/journal.pone.0291678 (PMC10511074; doi:10.1371/journal.pone.0291678)
Supplement: S1 Table — The hazard is calculated as a function of time since vaccination by pooling across the weekly vaccination cohorts with the same time since vaccination (i.e. cohorts with the same color have same number of weeks since vaccination). This is an example from MMWR week 3 2022 to MMWR week 11 in 2022. (DOCX) [file pone.0291678.s001.docx]

|  |  |  |  |  | **MMWR Week infected** | | |  |  |
| --- | --- | --- | --- | --- | --- | --- | --- | --- | --- |
| **MMWR Week Reaching ≥14 Days After Completing Vaccination** | 2022-03 | 2022-04 | 2022-05 | 2022-06 | 2022-07 | 2022-08 | 2022-09 | 2022-10 | 2022-11 |
| 2022-03 | 2 | 3 | 4 | 5 | 6 | 7 | 8 | 9 | 10 |
| 2022-04 |  | 2 | 3 | 4 | 5 | 6 | 7 | 8 | 9 |
| 2022-05 |  |  | 2 | 3 | 4 | 5 | 6 | 7 | 8 |
| 2022-06 |  |  |  | 2 | 3 | 4 | 5 | 6 | 7 |
| 2022-07 |  |  |  |  | 2 | 3 | 4 | 5 | 6 |
| 2022-08 |  |  |  |  |  | 2 | 3 | 4 | 5 |
| 2022-09 |  |  |  |  |  |  | 2 | 3 | 4 |
| 2022-10 |  |  |  |  |  |  |  | 2 | 3 |
| 2022-11 |  |  |  |  |  |  |  |  | 2 |

**S1 Table.** The number of weeks since vaccination is categorized using the duration between the week of vaccination to the week of infection. The hazard is calculated as a function of time since vaccination by pooling across the weekly vaccination cohorts with the same time since vaccination (i.e. cohorts with the same color have same number of weeks since vaccination). This is an example from MMWR week 3 2022 to MMWR week 11 in 2022
