# Supplementary material for: Application of a life table approach to assess duration of BNT162b2 vaccine-derived immunity by age using COVID-19 case surveillance data during the Omicron variant period
Source: PLoS One. 2023 Sep 20;18(9):e0291678. doi: 10.1371/journal.pone.0291678 (PMC10511074; doi:10.1371/journal.pone.0291678)
Supplement: S2 Table — (DOCX) [file pone.0291678.s002.docx]

**S2 Table. Completed STROBE checklist for observational study and analysis plan information**

|  | | Item No. | | Recommendation | Page  No. | | Relevant text from manuscript |
| --- | --- | --- | --- | --- | --- | --- | --- |
| **Title and abstract** | | 1 | | (*a*) Indicate the study’s design with a commonly used term in the title or the abstract | 4 | | surveillance, life table |
|  |  |  |  | (*b*) Provide in the abstract an informative and balanced summary of what was done and what was found | 4 | | This information is stated in the study abstract (study objective described, method and results described) |
| Introduction | | | | | | |  |
| Background/rationale | | 2 | | Explain the scientific background and rationale for the investigation being reported | 6 | | Rationale and existing literature are stated in the “Introduction” section |
| Objectives | | 3 | | State specific objectives, including any prespecified hypotheses | 6-7 | | A statement at the end of the introduction specifies the specific goals and objectives.  “This analysis uses hazard rates of SARS-CoV-2 infection estimated from surveillance and vaccine administrative data to indirectly investigate the duration of COVID-19 vaccine protection over time and by age group” |
| Methods | | | | | | |  |
| Study design | | 4 | | Present key elements of study design early in the paper | 7,9 | | Study design is stated in the first subsection of Methods. Key elements are all described in the methods.  “We analyzed reported numbers of SARS-CoV-2 infections by age group (5–11, 12–17, 18–49, 50–64, ≥65 years of age) from 22 U.S. jurisdictions (~53% of the U.S. population) with routine linkages between COVID-19 case surveillance and immunization information system (IIS) data reported to CDC during January 16, 2022 – May 28, 2022 through two different mechanisms .”  “We conducted a period cohort life table analysis” |
| Setting | | 5 | | Describe the setting, locations, and relevant dates, including periods of recruitment, exposure, follow-up, and data collection | 7,8 | | Setting, contexts, dates of inclusion, are fully described in the “Material methods” section under “COVID-19 case data by vaccination status” and “Population data by vaccination status” sections page 7 and 8. |
| Participants | | 6 | | (*a*) *Cohort study*—Give the eligibility criteria, and the sources and methods of selection of participants. Describe methods of follow-up  *Case-control study*—Give the eligibility criteria, and the sources and methods of case ascertainment and control selection. Give the rationale for the choice of cases and controls  *Cross-sectional study*—Give the eligibility criteria, and the sources and methods of selection of participants | 7,8 | | These data are neither a cohort, case-control, or randomized control study. These data are a secondary analysis using case counts aggregated by age during the period of January 16, 2022–May 28, 2022 over 22 states from each state’s surveillance system. The study data are fully described in the “Material Methods” section under “COVID-19 case data by vaccination status” and “Population data by vaccination status” sections page 7 and 8 |
|  |  |  |  | (*b*) *Cohort study*—For matched studies, give matching criteria and number of exposed and unexposed  *Case-control study*—For matched studies, give matching criteria and the number of controls per case |  | | Not applicable |
| Variables | | 7 | | Clearly define all outcomes, exposures, predictors, potential confounders, and effect modifiers. Give diagnostic criteria, if applicable | 7-10 | | These are fully described in the materials and method section pages 7-10.  Outcome: Time from the date a person reaches ≥14 days after completing primary vaccination of BNT162b2 vaccine to the date of a SARS-CoV-2 positive test or the end of the study period.  Independent Variable: 5–11, 12–17, 18–49, 50–64, ≥65 years of age  Exposure: vaccination status as described in “Material Methods”.  There were no other variables available for exposures, predictors, potential confounders, and effect modifiers |
| Data sources/ measurement | | 8* | | For each variable of interest, give sources of data and details of methods of assessment (measurement). Describe comparability of assessment methods if there is more than one group | *7-10* | | These are fully described in the “Material Methods” section pages 7-10. |
| Bias | | 9 | | Describe any efforts to address potential sources of bias | 10-11, 19-20 | | A sensitivity analysis is described to address the fact “we are unable to directly account for immunity from prior SARS-CoV-2 infection using our surveillance data, we incorporated external data from repeated seroprevalence studies to adjust for the prevalence of prior infection among the unvaccinated as part of a sensitivity analysis.” In addition, limitations of using surveillance data are described in the “Discussion” section. |
| Study size | | 10 | | Explain how the study size was arrived at | 7-8 | | Using all case counts aggregated by age during the period of January 16, 2022–May 28, 2022 over 22 states from each state’s surveillance system and See Table 1. Unvaccinated estimated as the number of persons each MMWR week by subtracting the cumulative number of vaccinated (all products) and partially vaccinated persons (all products) from the respective population totals for each jurisdiction and age group. |
| Continued on next page Quantitative variables | 11 | | Explain how quantitative variables were handled in the analyses. If applicable, describe which groupings were chosen and why | | 7 | 5–11, 12–17, 18–49, 50–64, ≥65 years of age. The vaccine was rolled out to particular age groups with older persons receiving priority, followed by younger persons. Among the last groups to receive authorization in our analysis 5-11 and 12-15. To maintain sufficient sample size of vaccinated cases for adults 18 and older during of January 16, 2022–May 28, 2022 - age groups were characterized as 18–49, 50–64, ≥65 which is consistent with the basis of many vaccine recommendations. | |
| Statistical methods | 12 | | (*a*) Describe all statistical methods, including those used to control for confounding | | 9-10 | Described in “Material Methods” under “Application of life tables to breakthrough surveillance data” | |
|  |  |  | (*b*) Describe any methods used to examine subgroups and interactions | | 7-8 | Described in “Material Methods”. As stated in abstract these data were used to examine potential waning of protection against SARS-CoV-2 infection for the Pfizer-BioNTech (BNT162b) primary vaccination series by age. There are no further subgroups or variables used in the analysis. | |
|  |  |  | (*c*) Explain how missing data were addressed | | 7-8 | Reliance was upon the data primarily received through a surveillance system from each state. These data were weekly aggregated case counts by product of the primary vaccination series (Pfizer-BioNTech (BNT162b)) and age. If age was missing then the case would not have been not included, if unknown vaccine product was unknown for either the 1st or 2^nd^ shot of the primary series then case would not have been included. As such these data underestimate COVID-19 rates. | |
|  |  |  | (*d*) *Cohort study*—If applicable, explain how loss to follow-up was addressed  *Case-control study*—If applicable, explain how matching of cases and controls was addressed  *Cross-sectional study*—If applicable, describe analytical methods taking account of sampling strategy | |  | See above | |
|  |  |  | (*e*) Describe any sensitivity analyses | | 10-11 | A sensitivity analysis is described to address the fact “we are unable to directly account for immunity from prior SARS-CoV-2 infection using our surveillance data, w | |
| Results | | | | | | | |
| Participants | 13* | | (a) Report numbers of individuals at each stage of study—eg numbers potentially eligible, examined for eligibility, confirmed eligible, included in the study, completing follow-up, and analysed | | 7-8, 10-11 | Reliance was upon the data primarily received through a surveillance system from each state. These data were weekly aggregated case counts by product of the primary vaccination series (Pfizer-BioNTech (BNT162b)) and age. Table 1 | |
|  |  |  | (b) Give reasons for non-participation at each stage | |  | Not applicable. Reliance was upon the data primarily received through a surveillance system from each state. | |
|  |  |  | (c) Consider use of a flow diagram | |  | Table 1 summarizes age by case | |
| Descriptive data | 14* | | (a) Give characteristics of study participants (eg demographic, clinical, social) and information on exposures and potential confounders | |  | Table 1 summarizes age by case | |
|  |  |  | (b) Indicate number of participants with missing data for each variable of interest | |  | Unavailable | |
|  |  |  | (c) *Cohort study*—Summarise follow-up time (eg, average and total amount) | | 7-8 | Using all case counts aggregated by age during the period of January 16, 2022–May 28, 2022 over 22 states from each state’s surveillance system—see Table 1 | |
| Outcome data | 15* | | *Cohort study*—Report numbers of outcome events or summary measures over time | | 7-8 | Using all case counts aggregated by age during the period of January 16, 2022–May 28, 2022 over 22 states from each state’s surveillance system—see Table 1 | |
|  |  |  | *Case-control study—*Report numbers in each exposure category, or summary measures of exposure | |  | *N/A* | |
|  |  |  | *Cross-sectional study—*Report numbers of outcome events or summary measures | |  | *N/A* | |
| Main results | 16 | | (*a*) Give unadjusted estimates and, if applicable, confounder-adjusted estimates and their precision (eg, 95% confidence interval). Make clear which confounders were adjusted for and why they were included | | 11-17 | Both unadjusted Both hazard rate ratios and the percent reduction in hazard rate of SARS-CoV-2 infection among the vaccinated as compared to the unvaccinated was provided in detail as well as standardized estimates. | |
|  |  |  | (*b*) Report category boundaries when continuous variables were categorized | | 7-8 | 5–11, 12–17, 18–49, 50–64, ≥65 years of age. | |
|  |  |  | (*c*) If relevant, consider translating estimates of relative risk into absolute risk for a meaningful time period | | 11-17 | Both hazard rate ratios and the percent reduction in hazard rate of SARS-CoV-2 infection among the vaccinated as compared to the unvaccinated was provided in detail. See Table 2 | |

| Other analyses | 17 | Report other analyses done—eg analyses of subgroups and interactions, and sensitivity analyses | 7-8,10-11 | No subgroups beyond age and vaccination status. Sensitivity analysis described in “Material Methods” |
| --- | --- | --- | --- | --- |
| Discussion | | | | |
| Key results | 18 | Summarise key results with reference to study objectives | 5,17 | First paragraph in the “Discission” and “Conclusions” in Abstract |
| Limitations | 19 | Discuss limitations of the study, taking into account sources of potential bias or imprecision. Discuss both direction and magnitude of any potential bias | 17-20 | Limitations and references are described in the “Discussion section”. |
| Interpretation | 20 | Give a cautious overall interpretation of results considering objectives, limitations, multiplicity of analyses, results from similar studies, and other relevant evidence | 17-20 | Limitations, described results from others studies and how they compare—are described in the “Discussion section” along with references. |
| Generalisability | 21 | Discuss the generalisability (external validity) of the study results | 19-20 | Addressed in so much as the limitations of surveillance data is described in the “Discussion” section |
| Other information | |  | | |
| **Funding** | **22** | **Give the source of funding and the role of the funders for the present study and, if applicable, for the original study on which the present article is based** | 8-9 | See “Ethics” section in “Material Methods”. “Funded by the Centers for Disease Control and Prevention” |

*Give information separately for cases and controls in case-control studies and, if applicable, for exposed and unexposed groups in cohort and cross-sectional studies.

**Note:** An Explanation and Elaboration article discusses each checklist item and gives methodological background and published examples of transparent reporting. The STROBE checklist is best used in conjunction with this article (freely available on the Web sites of PLoS Medicine at http://www.plosmedicine.org/, Annals of Internal Medicine at http://www.annals.org/, and Epidemiology at http://www.epidem.com/). Information on the STROBE Initiative is available at www.strobe-statement.org.
