# Supplementary figures and images for: Application of a life table approach to assess duration of BNT162b2 vaccine-derived immunity by age using COVID-19 case surveillance data during the Omicron variant period
Source: PLoS One. 2023 Sep 20;18(9):e0291678. doi: 10.1371/journal.pone.0291678 (PMC10511074; doi:10.1371/journal.pone.0291678)

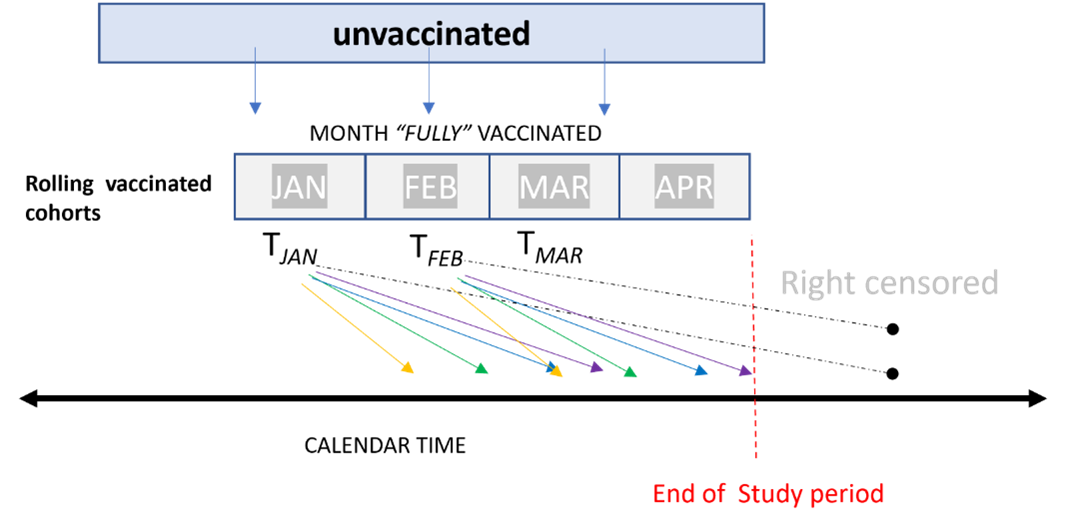

Supplement: S1 Fig — The solid lines with arrows indicate subjects in vaccination who subsequently had a positive SARS-Cov-2 test at various points in calendar time. The dotted lines subjects in the vaccination cohort without an observed SARS-Cov-2 test (right-censored). Lines with the same color have the same duration since vaccination to an observed SARS-Cov-2 test. These subjects are pooled to display an analysis with a single common hazard function across the cohorts with time since vaccination as the x-axis. (TIF) [file pone.0291678.s003.tif]

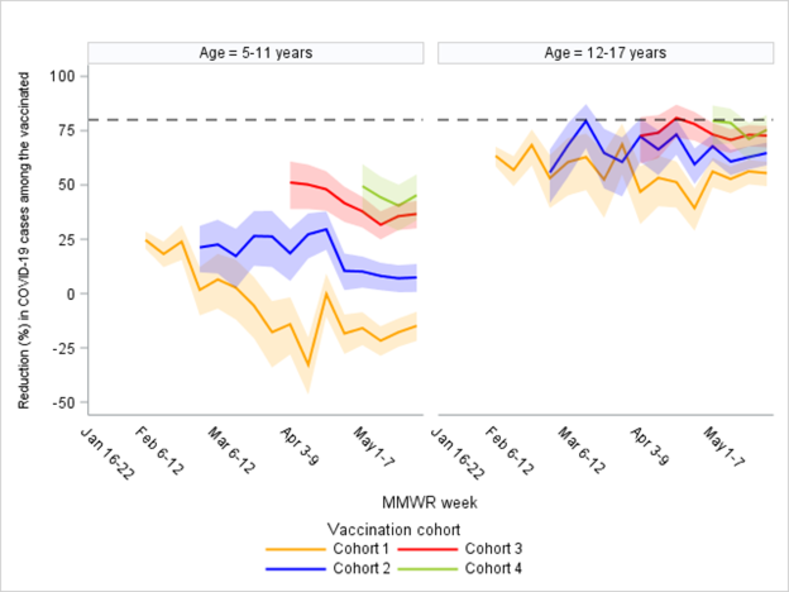

Supplement: S2 Fig — Black dashed reference line plotted at a VE of 80%. (TIF) [file pone.0291678.s004.tif]

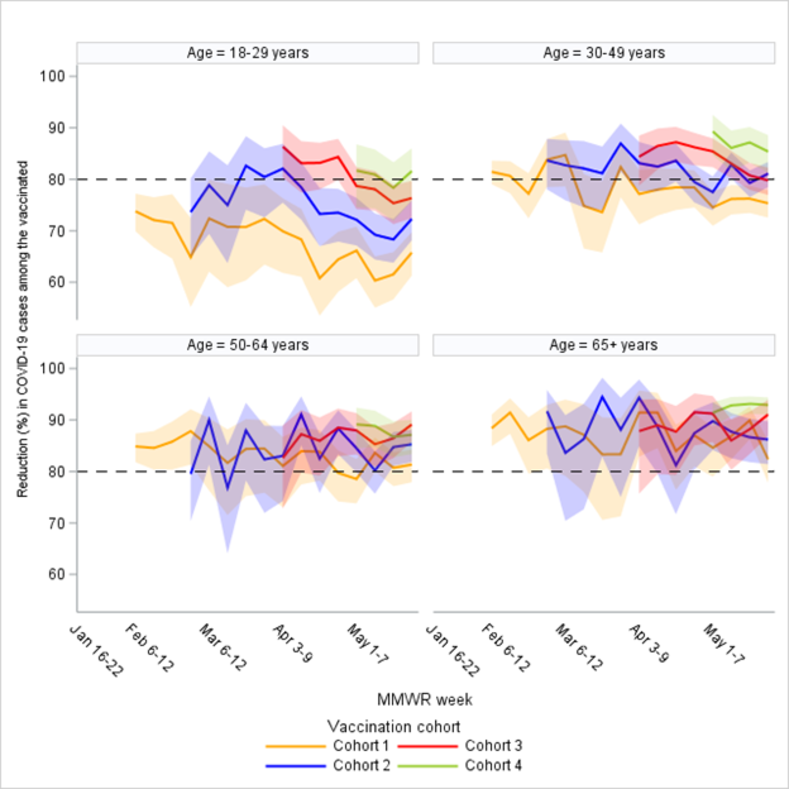

Supplement: S3 Fig — Vaccination cohorts 1–4 reached ≥14 days after vaccination with a Pfizer-BioNTech primary series during: January 16–February 5, February 6–26, February 27–April 2, April 3–30, 2022, respectively. Black dashed reference line plotted at a VE of 80%. (TIF) [file pone.0291678.s005.tif]

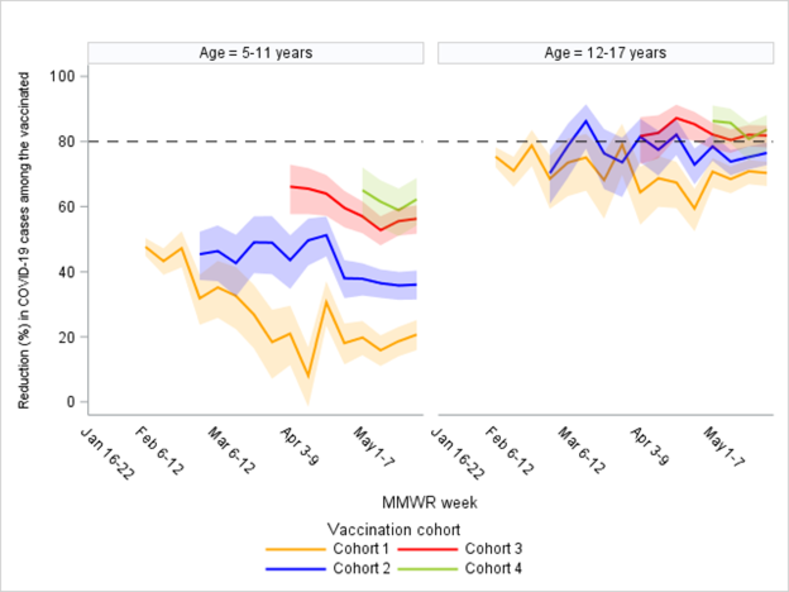

Supplement: S4 Fig — Vaccination cohorts 1–4 reached ≥14 days after vaccination with a Pfizer-BioNTech primary series during: January 16–February 5, February 6–26, February 27–April 2, April 3–30, 2022, respectively. For the sensitivity analysis, the total number unvaccinated at the beginning of the study period (week of January 16, 2021) was adjusted by a crude estimate of the potential difference in protection from prior infection by vaccination status using the difference in estimated SARS-CoV-2 seroprevalence by age group for September 2021 to January 2022 for ages 12–17 years and between November 2021 to January 2022 for 5–11 years [18], based on the later vaccine authorization for this age group. Black dashed reference line plotted at a VE of 80%. (TIF) [file pone.0291678.s006.tif]

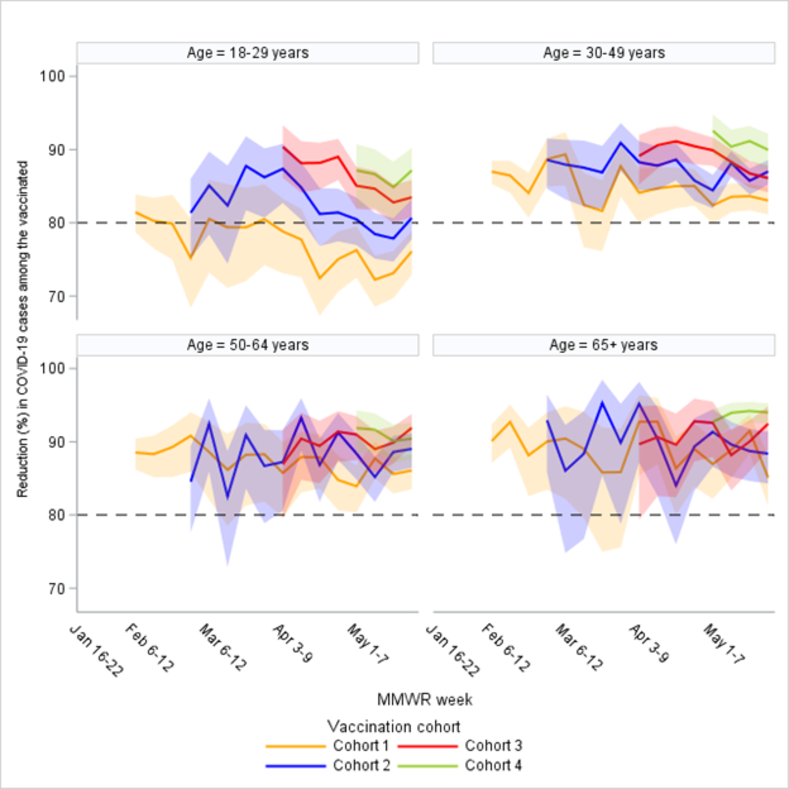

Supplement: S5 Fig — For the sensitivity analysis, the total number unvaccinated at the beginning of the study period (week of January 16, 2021) was adjusted by a crude estimate of the potential difference in protection from prior infection by vaccination status using the difference in estimated SARS-CoV-2 seroprevalence by age group for September 2021 to January 2022 for ages ≥18 years [18]. Black dashed reference line plotted at a VE of 80%. (TIF) [file pone.0291678.s007.tif]
