# Supplementary material for: Application of a life table approach to assess duration of BNT162b2 vaccine-derived immunity by age using COVID-19 case surveillance data during the Omicron variant period
Source: PLoS One. 2023 Sep 20;18(9):e0291678. doi: 10.1371/journal.pone.0291678 (PMC10511074; doi:10.1371/journal.pone.0291678)
Supplement: Attachment — Submitted filename: Response to Reviewers.docx [file pone.0291678.s008.docx]

**RE: PONE-D-23-06056**

**Application of a life table approach to assess duration of BNT162b2 vaccine-derived immunity by age using COVID-19 case surveillance data during the Omicron variant period**

*Sternberg et al.*

**We thank the reviewers for the helpful comments. Please note that the line numbers indicated below in our responses refer to the unmarked manuscript.**

**Editorial Office Review:**

***************************************

1. Please ensure that your manuscript meets PLOS ONE's style requirements, including those for file naming. The PLOS ONE style templates can be found at

<https://journals.plos.org/plosone/s/file?id=wjVg/PLOSOne_formatting_sample_main_body.pdf>

Response: **We have confirmed these requirements.**

and

<https://journals.plos.org/plosone/s/file?id=ba62/PLOSOne_formatting_sample_title_authors_affiliations.pdf>

Response: **Author affiliations have been fixed to ensure Affiliation footnotes appear in numerical order at first mention. Initials of corresponding author added after email.**

2. We note that you have stated that you will provide repository information for your data at acceptance. Should your manuscript be accepted for publication, we will hold it until you provide the relevant accession numbers or DOIs necessary to access your data. If you wish to make changes to your Data Availability statement, please describe these changes in your cover letter and we will update your Data Availability statement to reflect the information you provide.

Response: **We will provide relevant accession numbers to data if accepted for publication.**

3. Please include captions for your Supporting Information files at the end of your manuscript, and update any in-text citations to match accordingly. Please see our Supporting Information guidelines for more information: <http://journals.plos.org/plosone/s/supporting-information>.

Response: **We have included captions for supporting Information files at the end of the manuscript and updated our in text citations to match style requirements.**

4. Please review your reference list to ensure that it is complete and correct. If you have cited papers that have been retracted, please include the rationale for doing so in the manuscript text, or remove these references and replace them with relevant current references. Any changes to the reference list should be mentioned in the rebuttal letter that accompanies your revised manuscript. If you need to cite a retracted article, indicate the article’s retracted status in the References list and also include a citation and full reference for the retraction notice.

Response: **We have reviewed to ensure it is complete. Changes to the reference list were made to adequately address the reviewers’ comments and have been noted the response to reviewers.**

**Comments from Reviewer 1:**

***************************************

Major critique
1. A key assumption for the life table analysis is that the COVID-19 risk among subjects who are censored does not differ from the COVID-19 risk among those whose events are observed. As such, a seemly decreased hazard ratio of unvaccinated to the vaccinated could be due to increased risk among those with observed events. Therefore, one explanation of the seemly phenomenon of faster decay of VE in the young group can be that the deviation from the assumption grew larger along time for the young group than the older group.

Response: **Like many other analyses that use survival analysis techniques the assumption of non-informative censoring is the only way one can develop a likelihood to draw inferences on the failure time distribution. Any paper using survival methods, whether they explicitly write out the assumption or not—are making this assumption. This is not a testable assumption. In this analysis we use a fixed time censoring scheme with May 28, 2022 as the end of study.  This type of design has been proven by Lagakos & Williams as a special case of the non-informative censoring model – in other words the assumption of non-informative censoring is not violated under this type of fixed time censoring scheme.**

**We modified the statement about the non-informative censoring and reference to clarify this point.**

**(lines 192-194) *“****While a key assumption for methods of survival analysis is non-informative censoring, we used a fixed time censoring design, which has been proven to adhere to this assumption [16].”*

**We added a NEW reference lines 437-438** [16] Lagakos SW, Williams JS. 1978. Models for censored survival analysis: a cone class of variable-sum models. Biometrika 65:181–89.

**The example the reviewer provides…. “one explanation of the seemly phenomenon of faster decay of VE in the young group can be that the deviation from the assumption grew larger along time for the young group than the older group” is not an example of non-informative censoring. The same right censoring time was used for everyone on May 28^th^ 2022 and this date was selected as a matter of convenience and is unrelated to the failure time distribution.**

Minor critique
2. The first paragraph of Materials & Methods: “from 22 U.S. jurisdictions … through two different mechanisms. Eight jurisdictions reported … 12 jurisdictions reported …”—Please clarify the separate descriptions of 8 and 12 jurisdictions corresponded to the stated “two different mechanisms” or not? If so, 8+12 equals to 20 instead of 22; if not so, please rephrase as it’s unclear and confusing here.

Response: **This has been corrected. The 22 states used for this analysis are listed in lines 138-139. Lines 141-142 have been corrected to read as “***Nine jurisdictions reported line-level data for all COVID-19 cases, including date of specimen collection, vaccine product, and dates of vaccination; 13 jurisdictions reported line-level data ………”*

3. The four cohorts, January 16–February 5, February 6–26, February 27–April 2, and April 3–30, include 20, 20, 34, and 27 days respectively. What’s the rational to divide these four cohorts unevenly?

Response: **We have modified sentence in the methods to address (lines 149-151):** *“**We defined four vaccination cohort periods (1, 2, 3, and 4) based on MMWR weeks that roughly corresponded to calendar months: January 16–February 5, February 6–26, February 27–April 2, or April 3–30. “*

**The National Notifiable Diseases Surveillance System (NNDSS) disease report data in MMWR weeks which do not line up exactly with calendar weeks. We wanted to create monthly cohorts corresponding to Jan, Feb, March, April. We did our best to** **balance the length of the cohort and aligning it into a calendar month. While one could have shifted some of the MMWR weeks into different cohorts to emphasize equal duration it would not have made a difference to the final analysis. Prior to grouping the cohorts into “monthly” cohorts the analysis was conducted so that vaccination cohorts were based on a two-week periods. These periods were all equal width and generated a similar pattern, shape, and message as the current cohorts. In presenting the results graphically and in tables – it seemed easier to digest the message with fewer cohorts.**

**Comments from Reviewer 2:**

***************************************

 Comment 1: Abstract Results are confusing.
In the abstract it is confusing and almost seems contradictory when you say:
• “The percent reduction in hazard rates for vaccinated compared with unvaccinated persons was lowest among children aged 5–11 years at 35.5% (95% CI: 33.3%, 37.6%) compared to the older adults, which ranged from 68.7%–89.6%.” but then
• “The largest declines were observed among children aged 5–11 years and 12–17 years, while the declines among those 18 years and older were more modest after 19 weeks.”
• Also, how is this last sentence: “Among those 18 and older, the percent reduction in hazard rates among vaccinated compared with unvaccinated ranged from 66%–81%.” different from the 68.7% to 89.6% you mentioned above? Response: **The results are rewritten but to address the specific question the 66-81% was at 19 weeks while the 68.7-89.6 was at 2 weeks post vaccination.**
I found these results confusing and seemingly contradicting.
Also would either report % decimals everywhere or nowhere and not mix.

Response: **We have reworded the results section in the abstract (lines 98-103)** *“The percent reduction in hazard rates for persons 2 weeks after vaccination with a Pfizer-BioNTech primary series compared with unvaccinated persons was lowest among children aged 5–11 years at 35.5% (95% CI: 33.3%, 37.6%) compared to the older age groups, which ranged from 68.7%–89.6%. By 19 weeks after vaccination, all age groups showed decreases in the percent reduction in the hazard rates compared with unvaccinated people; with the largest declines observed among those aged 5–11 and 12–17 years and more modest declines observed among those 18 years and older.”*

**We are reporting 1 decimal place for the percents consistently in the abstract.**

Comment 2: I think this could be worded more clearly “differences in prior infection-derived immunity by vaccination status” … do you mean that unvaccinated people are more likely to derive infection-induced immunity over time? If so, I would clarify as the language is non-specific.

Response: **In order to address comment 11 from reviewer 2 and due to the word limit in the abstract the sentence referenced by the reviewer has been removed and replaced with the following (lines 105-107) *“****The decline in vaccine protection against SARS-CoV-2 infection observed in this study is consistent with other studies and demonstrates that national case surveillance data were useful for assessing early signals in age-specific waning of vaccine protection during the initial period of SARS-CoV-2 Omicron variant predominance.”*

Comment 3: Please cite Tartof et al. in Lancet for this sentence [as well], which was really the first paper that outlined this: “A study during the Delta period showed evidence of waning protection against infection but strong protection against death 6 months after vaccination”

Response: **We have added a NEW reference as citation # 4 at line 122 in the Introduction and line 317 in the Discussion.**

**The NEW reference at lines 401-403** **as** Tartof SY, Slezak JM, Fischer H, Hong V, Ackerson BK, Ranasinghe ON et al. Effectiveness of mRNA BNT162b2 COVID-19 vaccine up to 6 months in a large integrated health system in the USA: a retrospective cohort study. Lancet. 2021 Oct 16;398(10309):1407-1416. doi: 10.1016/S0140-6736(21)02183-8. PMID: 34619098.

Comment 4: Are results similar if you excluded all the sites where you had to make the unvaccinated correction factor? May be a worthwhile sensitivity analysis perform and at least mention that this did not majorly influence the results, even if only about 5% of overall data.

Response: **As stated in the text, this continuity correction impacted those 65 and older in only 5 of the 22 jurisdictions during our study period. The assumption of 95% vaccine coverage for this age group is reasonable see** [CDC COVID Data Tracker: Vaccinations in the US](https://covid.cdc.gov/covid-data-tracker/#vaccinations_vacc-people-booster-percent-pop5).

**To address the reviewer, but not to add another figure to the final paper—we included the results of a sensitivity analysis using a lower the vaccination coverage for 5.3% of the combinations of 17 MMWR weeks, 7 age groups, and 22 jurisdictions which required the correction. As seen below in the figure below, a threshold of 85% vaccination coverage did not impact any age groups except the 65 and older. With this change - the 65 and older standardized reduction in the case hazard rate would have been more similar to those 50-64 year old (see graph below). To assume anything lower than 85% is not consistent with what has been reported for vaccination coverage for this group. Comparing this graph to Figure 3 in the paper, the difference is negligible.**

**The continuity correction described has been used in other published papers that use COVID19 case and vaccination surveillance data – we have also added a reference number in line 172 to reference [9] which was already referenced in the original paper.**

**In addition, we added the following sentence at lines 174-176** *“A sensitivity analysis (not shown) showed this continuity correction to not have a substantive impact on the reported findings in this paper.”*

**Figure. Standardized percent reduction in hazard rate for COVID-19 cases — 22 U.S. jurisdictions, January 16 to May 28, 2022 using 15% as a continuity correction instead of 5% (.e. 85% vaccination coverage instead of 95%)**

Comment 5: Did you test this key assumption in any way? Can you reassure the reader this is probably not a major issue? “A key assumption for the life table analysis is that the COVID-19 risk among subjects who are 191 censored does not differ from the COVID-19 risk among those whose events are observed (known as non informative censoring) [15].”

Response: **Like many other analyses that use survival analysis techniques the assumption of non-informative censoring is the only way one can develop a likelihood to draw inferences on the failure time distribution. Any paper using survival methods, whether they explicitly write out the assumption or not—are making this assumption. This is not a testable assumption. In this analysis we use a fixed time censoring scheme with May 28, 2022 as the end of study.  This type of design has been proven by Lagakos & Williams as a special case of the non-informative censoring model – in other words the assumption of non-informative censoring is not violated under this type of fixed time censoring scheme.**

**We modified the statement about the non-informative censoring and reference to clarify this point.**

**– lines 192-194 *“****While a key assumption for methods of survival analysis is non-informative censoring, we used a fixed time censoring design, which has been proven to adhere to this assumption [16].”*

**We added a NEW reference lines 437-438 [16]** Lagakos SW, Williams JS. 1978. Models for censored survival analysis: a cone class of variable-sum models. Biometrika 65:181–89.

Comment 6: “Since there are no seroprevalence data available by vaccination status, we assumed that infections preferentially occurred in unvaccinated people during periods after the vaccine had been authorized.” This is probably true, and your method of handling this was very clever, but did you model the impact if you assumed equal likelihood of infection between the two groups? This would model a world if vaccine did not significantly interrupt transmission or infection, but lessened severity instead. Would be an important sensitivity analysis perhaps.

Response: **If the prior infection between the two groups is the same there would be no impact on the VE after this adjustment because VE is a relative measure between the vaccinated and unvaccinated. The sensitivity analysis is intended to get a glimpse of the results if there is differential immunity between the two groups which as the reviewer comments is likely true given the vaccine effectiveness noted from the randomized clinical trials.**

Comment 7: How do explain the potential negative reductions in 5-11 year-olds in Table 2? This will probably be picked up by several readers?

Response: **One explanation are potential differences in natural immunity by vaccination status which is the reason behind the sensitivity analysis using age specific seroprevalence. At the time of this revision several studies have reported negative VE during Omicron (case surveillance (Dorabawila et al) as well as TNDs (Buchan et al, Hansen at al, Altarawneh et al, Andrews et al, Chemaitelly et al, Ranzanai et al). Given that results from well-designed randomized clinical trials support the efficacy of the BNT162b2 COVID-19 vaccine, a likely explanation for these observed negative VE is bias and confounding. While test-negative designs may be better suited to control for selection biases and confounding variables (such as testing, case reporting and health-seeking behaviors) than vaccine coverage and case surveillance data, neither design is immune to potential bias as noted by the several TND with negative VEs. A recent paper by Bodner et al discusses various biases that may yield false negative VE estimates. We have added some language about the negative VE as well as the NEW Bodner reference as [33]**

**Lines 334-336** “*Estimates of prior infection are higher in younger age groups and likely also differ by vaccination status [19], a potential bias that may explain the negative VE observed among children 5-11 years [33], and which we tried to demonstrate through a sensitivity analysis*.”

**NEW Reference added at Lines 490-492 [33]** Bodner K, Irvine MA, Kwong JC, Mishra S. Observed negative vaccine effectiveness could be the canary in the coal mine for biases in observational COVID-19 studies. Int J Infect Dis. 2023 Jun;131:111-114. doi: 10.1016/j.ijid.2023.03.022. PMID: 36990200.33.

Comment 8: This entire analysis is based on cases. How do you deal with differences in healthcare seeking / testing behavior which may be a major factor, especially for the endpoint of COVID-19 cases? Can you perform this same analysis on hospitalization?

Response: **The use of national surveillance data to examine questions about real-time vaccine impact continues to be integral part of an on-going surveillance system, despite the standard limitations of surveillance systems, namely, underreporting and lack of representativenes.**

**We have added a sentence in the abstract to re-emphasize the scientific value of this approach lines 105-107. We address the limitations that we are unable to account for healthcare seeking / testing behavior by age and vaccination status—see lines 362-365**

“*We were unable to account for differences in underlying conditions, testing practices, prior infection, exposure risk, or prevention behaviors by vaccination status, age, geography, and over time; current results were influenced by the circulating Omicron variant, the relatively high prevalence of prior infection among children, and differences in testing practices and vaccine seeking behaviors compared to 2021.*”

**Hospitalization data were not reported from the sites involved in this study, so we are unfortunately unable to use this endpoint.**

Comment 9: What does the red dashed line in figure 4 at 80% represent?

Response: **It is a benchmark. We have added the following language to the plot title or legend where relevant "***A dashed reference line is plotted at a VE of 80%.*” **E.g Line 306**

Comment 10: Did school closures during the study period potential impact any of the findings? Is there any evidence that 5-11 year-olds kept more social interaction through daycare/after school programs compared to 12-17 year olds who could likely stay home on their own? Perhaps an explanation of the 5-11 year old findings? Are there other larger social influences besides the lower dose that could explain this? Might be worth contextualizing.

Response: **A study conducted in California during 2020- 2021, before our study period, suggested that 5-12 years and 13-17 years did not have different contact patterns (ref: Andrejko, K.L., Head, J.R., Lewnard, J.A. et al. Longitudinal social contacts among school-aged children during the COVID-19 pandemic: the Bay Area Contacts among Kids (BACK) study. BMC Infect Dis 22, 242 (2022). https://doi.org/10.1186/s12879-022-07218-4)**

**If this study can be generalized than the higher seroprevalence has been observed among 5-11 years as compared to 12-17 may be instead due to the later approval of the vaccine for 5-11 year olds compared 12-17 year olds, especially if the contact rate were similar. We have added the sentence 338-341.** *“Higher seroprevalence has been observed among children 5–11 years, likely due in part to this being among the last groups to be authorized to receive the vaccine. Additionally, school-based testing practices are not well-documented but could have resulted in greater rates of detection of mild/asymptomatic cases in both vaccinated and unvaccinated children.”*

Comment 11: I mentioned before the impact of testing proclivity. You mention very clearly in the results that “We were unable to account for differences in underlying conditions, testing practices, prior infection, or prevention behaviors by vaccination status, age, geography, and over time; current results were influenced by the circulating Omicron variant, the relatively high prevalence of prior infection among children, and differences in testing practices and vaccine seeking behaviors compared to 2021.” I guess the question many will ask, is—if so many limitations of this life-table approach—why even perform it? Doesn’t it have way more limitations than adjusted analyses or TND approaches that can help condition on testing? I think this should be better explained by the authors. What does this approach add? Don’t we know vaccines wane after about 2-3 months against mild illness already? What does this approach and paper clearly add? I would spell this out a little better—because it just feels like a crude analysis when others have already reported better adjusted results.

Response: **The reviewer’s comment is well taken. To clarify--the limitations are not due to the lifetable analysis, but inherent to surveillance data. We have noted the limitations of our surveillance data in lines 362-365. In addition, we noted the value of these data in lines 342-345 as well as with the new sentence added to the abstract – lines 105-107.**

**There is value in monitoring and reporting results from national surveillance data to generate early signals to detect changes in rate ratios by vaccination status over time which may indicate changes in protection due to emerging variants and waning immunity for evaluation with robust VE studies before these studies can be conducted and completed.** **The value of this paper is describing a statistical approach that can be use somewhat real-time data from disparate streams of case surveillance data (both aggregated and line listed) to investigate pressing questions of public health. At the time when this method was used there was an urgent need for answers to questions about *observed* vaccine effectiveness and whether there was evidence of waning. The challenge at that time was to develop an approach that could use both line-listed and aggregated case counts data on vaccine uptake over the MMWR weeks. The value in the approach is to demonstrate that this can be done by defining various cohorts and assessing their hazard function (as estimated by lifetable) over time. Other surveillance programs may find this useful to implement.**

Comment 12: Finally, although the vaccine does wane, with the exception of the 5-11 group… it seems like the vaccine continues to provide protection through week 19 and likely beyond. Is this consistent with TNDs that have looked at VE against infection/symptomatic disease beyond 3 or 4 months? How do you characterize this? I would highlight you still see protection beyond 19 weeks in this study… despite some waning—because that is what you find. But contextualize with other reports.

Response: **We have changed wording in the discussion to frame the waning immunity findings to highlight as suggested (lines 312-313) “***At 19 weeks after vaccination, decreased protection against infection was observed for all age groups, residual vaccine protection was observed for ages 12 and older.***”**

**We have added some language to contextualize with other studies the paragraph with lines 314-326.**

**Specifically, we added 2 new references cited in the following sentences: Lines 315-317** *“Vaccine protection against severe COVID-19 outcomes was not evaluated, but has been shown elsewhere to be more robust and durable than protection against infection and symptomatic disease [3, 4, 20-22].”*

**We reference a paper that was already part of our references with the new sentence at lines 322-323 “***A meta-review showed several other VE studies have shown residual protection against infection up to 5 months [23].”*

**NEW references 20-21 lines 444-449**

20. Andrews N, Tessier E, Stowe J, Gower C, Kirsebom F, Simmons R et al. Duration of Protection against Mild and Severe Disease by Covid-19 Vaccines. N Engl J Med. 2022 Jan 27;386(4):340-350. doi: 10.1056/NEJMoa2115481. PMID: 35021002.

21. Ferdinands JM, Rao S, Dixon BE, Mitchell PK, DeSilva MB, Irving SA et al. Waning of vaccine effectiveness against moderate and severe covid-19 among adults in the US from the VISION network: test negative, case-control study. BMJ. 2022;379:e072141. doi: 10.1136/bmj-2022-072141. PMID: 36191948.
